# Editorial: The impact of COVID-19 on internet addiction, suicidal behavior, and study behavior in adolescents in various cultural contexts

**DOI:** 10.3389/fpsyt.2024.1375244

**Published:** 2024-02-06

**Authors:** Mao-Sheng Ran, Yunyu Xiao, Hans Rohlof

**Affiliations:** ^1^ Mental Health Center, West China Hospital, Sichuan University, Chengdu, Sichuan, China; ^2^ Weill Cornell Medicine, Cornell University, New York, NY, United States; ^3^ Utrecht University, Utrecht, Netherlands

**Keywords:** COVID-19, internet addiction, suicidal and study behavior, adolescents, cultural contexts

The coronavirus disease 2019 (COVID-19) pandemic and related quarantine have affected mental health problems, especially among adolescents. Social distancing, school closures, and separation from families were associated with increased prevalence of internet addiction, suicidal behavior, and poor learning abilities among youth populations. Given adolescents are in critical periods of neurodevelopment, they may be more vulnerably impacted by the COVID-19 pandemic, vaccine uptake strategies, quarantine policies and measures, health services and schooling arrangements during and after the pandemic. While it is crucial to explore the long-term impact of COVID-19 on adolescent mental health and behaviors, there is still limited data on internet addiction, suicidal behavior, and study behavior in adolescents, especially across in various cultural contexts.

This editorial on the Research Topic synthesizes insights from seven pivotal studies, shedding light on the pandemics extensive impact of COVID-19 pandemic on adolescent mental health, with a focus on internet addiction, suicidal behavior, and academic engagement across various cultural contexts.

For internet addiction (IA), the study by Deng et al., conducted among students in colleges and universities (n=22,605) in Sichuan, China, showed that most students (66.0%) had internet addiction after the lifting of COVID-19 restrictions. Students with IA were more likely to have depression symptoms, anxiety symptoms, insomnia, and lifetime suicidal ideation, which is consistent with previous studies (1). A significant IA-by-academic satisfactory-interaction on mental health was identified. The finding of this study reveals that IA is significantly linked to mental health issues such as depression, anxiety, and suicidal ideation. The COVID-19 pandemic has had both acute and long-term psychological impact on children and adolescents (2). Moreover, IA and academic satisfaction have interactive impacts on mental health problems.

Another study by Petrovićet al., conducted in Croatia during the COVID-19 pandemic, showed that young adults had increased problematic internet use (60.8%) and problematic online gaming (25.9%). Problematic internet use was significantly associated with more depression symptoms, which is consistent with previous studies (3).

The issue of self-harm in adolescents is brought to the fore by Do et al. in Korea, where a noticeable increase in the usage of “self-harm” in social media texts was observed during the second wave of COVID-19 (from August to September 2020). Korean adolescents were more likely to share psychological challenges such as self-harm with friends rather than their parents, which is consistent with previous studies (4). This trend also aligns with adolescents preferring to share psychological challenges with peers rather than parents. This study suggests that it is necessary to support these adolescents in the prolonged pandemic era (2).

For the suicide behavior, the study in Japan of Dat et al. indicated that individuals with low self-esteem reported high psychological distress, which may further lead to hopelessness and heightened suicidal ideation. Psychological distress exerted a greater impact on suicidal ideation during COVID-19 pandemic, which is consistent with previous studies (2). This study suggests that self-esteem, hopelessness, and psychological distress can help elucidate the development of suicidal ideation. It should be helpful to target these factors for suicide prevention.

Another study on non-suicidal self-injury (NSSI) in adolescents in China by Liu et al. identified depression, low self-esteem, excessive internet use, and sleep disturbance as significant risk factors. These findings highlight the intricate relationship between self-esteem, online behavior, and mental health in the context of NSSI.

Furthermore, the study by Song et al. on Chinese middle and high school students (n=60268) revealed that over 60% reported pandemic-related academic disruptions, with a substantial number experiencing a decline in academic performance (41.2%). There was a significant association between academic performance change and mental health problems, which is consistent with previous studies (5). Improved academic performance was as protective factor for depression, and declined academic performance was a risk factor for depression and anxiety. Being COVID-19-infected, having family members being infected, having quarantine experience, and with COVID-19-related stigma were risk factors for depression and anxiety. This study indicates that academic studies and the mental health status of middle and high school students in China have been negatively impacted by the COVID-19 pandemic, even after the lifting of COVID-19 restrictions. This study has shed light on the important relationship between academic performance and mental health of adolescents.

In conclusion, studies in this section suggest that there is long-term impact of COVID-19 pandemic on adolescents mental health and behaviors, especially internet addiction, suicidal behavior and study behavior. These studies collectively illustrate the profound and long-term impact of the COVID-19 pandemic on adolescent mental health and suicidal behaviors. The pandemic has not only exacerbated issues like internet addiction and suicidal tendencies but also significantly disrupted academic pursuits. Thus, it is crucial to develop tailored health policies and interventions to help adolescents to cope with the emergency, as well as to address the specific needs and challenges of these adolescent and young students. Educational institutions and policymakers must integrate mental health support with academic strategies, acknowledging the critical interplay between academic performance and psychological well-being. Future research should aim to further elucidate these relationships and explore effective intervention strategies, particularly in both Eastern and Western contexts (6). These findings serve as a clarion call for concerted efforts in addressing the nuanced and profound impact of the COVID-19 pandemic on adolescent mental health globally. By fostering a supportive and responsive environment, we can mitigate these challenges and promote healthier, more resilient youth populations.

## Author contributions

MR: Writing – original draft, Writing – review & editing. YX: Writing – review & editing. HR: Writing – review & editing.
